# Addressing critical knowledge and capacity gaps to sustain CRVS system development

**DOI:** 10.1186/s12916-020-01523-y

**Published:** 2020-03-09

**Authors:** Tim Adair, Nicola Richards, Avita Streatfield, Megha Rajasekhar, Deirdre McLaughlin, Alan D. Lopez

**Affiliations:** Melbourne School of Population and Global Health, The University of Melbourne, Carlton, Victoria 3053 Australia

**Keywords:** Capacity-building, Civil registration and vital statistics, Courses, Evidence, Fellowship, Sustainability, Training, Quality

## Abstract

**Background:**

Improving civil registration and vital statistics (CRVS) systems requires strengthening the capacity of the CRVS workforce. The improvement of data collection and diagnostic practices must be accompanied by efforts to ensure that the workforce has the skills and knowledge to assess the quality of, and analyse, CRVS data using demographic and epidemiological techniques. While longer-term measures to improve data collection practices must continue to be implemented, it is important to build capacity in the cautious use of imperfect data. However, a lack of training programmes, guidelines and tools make capacity shortages a common issue in CRVS systems. As such, any strategy to build capacity should be underpinned by (1) a repository of knowledge and body of evidence on CRVS, and (2) targeted strategies to train the CRVS workforce.

**Main text:**

During the 4 years of the Bloomberg Philanthropies Data for Health (D4H) Initiative at the University of Melbourne, an extensive repository of knowledge and practical tools to support CRVS system improvements was developed for use by various audiences and stakeholders (the ‘CRVS Knowledge Gateway’). Complementing this has been a targeted strategy to build CRVS capacity in countries that comprised two approaches – in-country or regional training and a visiting Fellowship Program. These approaches address the need to build competence in countries to collect, analyse and effectively use good quality birth and death data, and a longer-term need to ensure that local staff in countries possess the comprehensive knowledge of CRVS strategies and practices necessary to ensure sustainable CRVS development.

**Conclusion:**

The Knowledge Gateway is a dynamic, useful and long-lasting repository of CRVS knowledge for countries and development partners to use to formulate and evaluate CRVS development strategies. Capacity-building through in-country or regional training and the University of Melbourne D4H Fellowship Program will ensure that CRVS capacity and knowledge is developed and maintained, facilitating improvements in CRVS data systems that can be used by policymakers to support better decision-making in health.

## Background

An essential element of any strategy to improve civil registration and vital statistics (CRVS) is to strengthen the capacity of the CRVS workforce. The complete registration of deaths and accurate ascertainment of the cause of death (COD) is reliant upon improving data collection and diagnostic practices, including efficient CRVS system design, accurate medical certification and COD coding, and the application of best practice and innovations in verbal autopsy (VA) to identify the CODs in low-resource settings. However, improvement of such skills in isolation is insufficient; it is equally important to ensure that the workforce has the skills and knowledge in data quality assessment and analysis of CRVS data using demographic and epidemiological techniques as a key input to support policy debates. Building capacity in the careful use of imperfect data is particularly important while longer-term measures to improve data collection practices are being implemented. These skills are essential for countries to strengthen and use vital statistics to monitor progress towards national and international health goals such as the Sustainable Development Goals, where cause-specific mortality data is needed for 7 of the 17 goals and 17 of their corresponding indicators alone [1].

Unfortunately, capacity shortages are common in CRVS systems because of the nascent scientific foundations of the discipline, limited face-to-face training programmes and, until recently, few accessible and easy-to-use guidelines and tools to increase knowledge in these topic areas. Within this context, it may be argued that any strategy to build essential capacity should be underpinned by two fundamental and complementary goals – (1) the creation of a lasting repository of knowledge and body of evidence on CRVS, including information on the drivers of successful (or unsuccessful) initiatives as well as a compilation of relevant, accessible and user-friendly tools and guidelines that countries can use, and (2) targeted strategies to train the future CRVS workforce, including short courses and longer-term fellowships, to ensure there is the requisite individual and institutional capacity to sustain CRVS systems development in countries. We expand on these two strategies below, reporting on the experience of the Bloomberg Philanthropies Data for Health (D4H) Initiative at the University of Melbourne (UoM).

### Building the evidence base

While global interest in improving CRVS systems has gradually strengthened given the monitoring requirements associated with global development initiatives such as ‘Health for All by the Year 2000’ and the Millennium Development Goals [2, 3], it was the 2007 ‘Who Counts?’ [4] *Lancet* series that stimulated a more urgent call to action to strengthen CRVS systems, crystallised ideas and challenges, and outlined why the global community should invest in improving basic health data on births and deaths as a matter of priority. From 2008 to 2013, the Health Information Systems Knowledge Hub at the University of Queensland developed tools and courses for building in-country capacity in CRVS systems, including rapid assessments of CRVS systems and assessing the quality of mortality statistics, both of which were promoted by the World Health Organisation [5–7]. Additionally, published studies have been useful in reporting on the features and shortcomings of national CRVS systems [8–12], or the quality and accessibility of vital statistics [13–16]. However, aside from these, there have been limited practical tools for countries to use to aid in the interpretation of data and the integration of findings into national planning agendas.

In response to the paucity of publicly available information on CRVS development strategies and outcomes, a central aim of the D4H Initiative has been to generate a repository of knowledge and evidence to fill clear gaps in available tools and guidelines, building on existing products (where available), based on the experiences of D4H countries^1^ with their CRVS system-strengthening efforts. Over 4 years, 150 products detailing outcomes of interventions, ranging from automated VA for community deaths, improving medical certification practices and assessing the quality of COD data, have been developed to help fill this knowledge gap. This repository of knowledge products is available online in the CRVS Knowledge Gateway Library [17], where knowledge resources are grouped into five thematic areas, namely (1) applying country experiences and knowledge; (2) improving death certification and mortality coding; (3) implementing VA; (4) strengthening CRVS systems; and (5) transforming data into information for action. These themes were chosen given their central role in building critical knowledge and capacity for CRVS system strengthening, specifically regarding the strengthening of systems for the generation and analysis of mortality statistics. An overarching focus across these five thematic areas was on innovation, best practice, country experiences and producing teaching materials to strengthen CRVS practices around data collection, data quality assessment and data analysis.

Having such a repository of CRVS knowledge, experiences, tools, guidelines, and monitoring and evaluation frameworks is critical in order to support country CRVS efforts. A key aim was to make country partners aware of the suite of resources available and how best to benefit from them, particularly the tools and guidelines designed for immediate use. The success of this approach is demonstrated in several countries, for example, Sri Lanka, who used the generic Handbook for Physicians on Cause of Death Certification [18] as the basis for their country-specific COD mobile application [19] and online medical certification training course. Similarly, Myanmar was able to use the Medical Certification of Cause of Death: Undergraduate Curriculum [20] contained in the Knowledge Gateway to develop a country-specific training module for undergraduate students on medical certification. Brazil also used the generic report template for the data quality assessment tool ANACONDA (Analysis of National Cause of Death for Action) [21],^2^ translated it into Portuguese, adapted it to their needs and integrated it into their routine COD reporting. Importantly, all the products were extensively tested and validated in multiple countries; for example, ANACONDA was modified based on application in several country or intercountry workshops.

The country-specific tailoring of these resources ensures that they are more easily embedded into national CRVS systems. This has been facilitated through extensive translations of core resources into primary D4H languages, including Chinese, Portuguese and Spanish – notwithstanding the number of resources that have also been adapted for specific in-country use, including Sinhalese and Burmese versions of the VA training resources. Several resources were also translated into French and Russian as part of regional training workshops, expanding the impact of these resources beyond the D4H countries.

### Training the future CRVS workforce

Unlike demography or epidemiology, CRVS is not an established academic discipline, with a consequent severe lack of educational resources on the topic. The D4H Initiative has been building the capacity of analysts in national statistical offices, ministries of health and registration agencies to apply cost-effective methods to generate reliable COD data for planning across a range of content areas, including medical certification, COD coding, VA and data quality assessments as well as building skills for data analysis, which are directly relevant to monitoring the burden of disease and progress with development goals. Capacity-building has comprised two approaches – in-country or regional training and a visiting Fellowship Program. These complementary approaches address the acute need to build the confidence and competence of the current CRVS workforce to collect, analyse and effectively disseminate good quality data, and a longer-term need to ensure CRVS systems development efforts in countries are supported by local staff with a comprehensive knowledge of CRVS strategies and best practices to ensure sustainability.

In acknowledging that knowledge products alone are unlikely to produce system-wide, sustainable change, the University of Melbourne has led the development and delivery of 10 technical courses on CRVS (Table 1). Each course has been developed with a specific training need in mind, mostly related to a specific CRVS intervention, with delivery methods and didactic approaches for each course dependent on the scope, target audience, and existing country capacities and needs. Preference was for adult learning approaches, hands-on examples and practical exercises, and use of local expertise and experiences whenever possible, including partnering with appropriate local institutions, such as the Philippines Statistical Authority, who have implemented a vast in-country training programme for the application of ANACONDA [21]. As the courses were intended to form part of a broader strategy to support CRVS system development in countries, many were delivered using a ‘training of trainers’ approach, thus building in-country capacity to implement ongoing and extensive training programmes in critical areas such as medical certification of COD. These training courses have been conducted in 18 D4H Initiative countries and regional training has been performed for UN and WHO agencies including participants from many other countries. These courses, together with the CRVS Knowledge Gateway, have led to substantial improvements in the scope and quality of mortality and COD statistics in D4H countries. For example, as of 2019, VAs have been conducted for over 20,000 community deaths in Bangladesh and 50,000 in Myanmar; prior to the Initiative, there was no reliable information on the causes of community deaths in these countries. Additionally, benefits of the D4H medical certification of COD training in Peru have been demonstrated, with results being used to further strengthen the training [22].

**Table 1:** Courses developed by Bloomberg Philanthropies Data for Health (D4H) Initiative at the University of Melbourne (UoM)

Course	Description
ANACONDA	Builds capacity and confidence in participants to use and apply results from the ANACONDA tool; participants learn how to assess mortality data quality using basic epidemiological and demographic concepts, and to interpret and apply the results
ANACONDA Plus	Expands on the concepts of the ANACONDA course, including how to adjust and analyse data to improve their value for policy
CRVS Bootcamp	Provides an overview of CRVS systems and the work taking place under UoM D4H, and often includes presentations by visiting Fellows
Data analysis and use (developed in partnership with Vital Strategies)^a^	Trains participants to effectively analyse and present CRVS data to provide evidence for policy and planning
Enterprise architecture, process mapping (developed in partnership with Swiss Tropical and Public Health Institute)	Introduces the basic principles of enterprise architecture business process mapping and helps participants develop the skills needed to apply this system analysis approach to CRVS systems
Estimating the completeness of registration	Improves participants’ ability to utilise a range of methods to estimate the completeness of birth and death registration as a means of generating reliable fertility and mortality measures from the CRVS system
ICD-10 coding for clerks and coders	This course is offered for mortality coding staff working in healthcare or civil registration offices that use the ICD-10 health classification system
Iris coding for experienced coders	This course is offered to meet the needs of mortality coding staff working in institutions where the automated coding software Iris is being introduced
Medical certification of COD	A ‘training of trainers’ programme for doctors on ICD-10-compliant death certification practices
VA	Covers the importance of VA for COD data, roles and responsibilities of VA interviewers and supervisors, ethics and sensitivity, the SmartVA questionnaire, and how to collect information using a tablet

^a^This course was developed with Vital Strategies, who were responsible for the Data Impact component of the D4H Initiative. This partnership built on the respective strengths of the two partners, namely the technical expertise of UoM and the data dissemination and use skills of Vital Strategies

*ANACONDA* Analysis of National Cause of Death for Action; *COD* cause of death; *CRVS* civil registration and vital statistics; *ICD-10* International Classification of Diseases and Related Health Problems – 10th revision; *VA* verbal autopsy.

A second related, but longer-term strategy, has been the purposeful development of in-country CRVS knowledge across the spectrum of data collection, assessment and use via the CRVS Fellowship Program at the University of Melbourne and Swiss Tropical and Public Health Institute. Typically, Fellows have spent 2–3 months engaged in intensive, one-on-one mentoring with technical specialists. The Program has been designed to build capacity in a sustainable manner beyond what can be achieved in training courses delivered to larger audiences; it is open to individuals working within CRVS systems of D4H countries and whose participation in the Program will help to enhance the sustainability of CRVS activities in their country. Fellows work on a project topic with direct application to CRVS activities in their country. To provide a good breadth of experience, Fellows also engage widely with other Fellows and D4H technical specialists on topics not necessarily directly related to their own area of speciality, participate in a 2-day CRVS ‘Bootcamp’ (Table 1) to learn about the wide range of CRVS interventions, and present on their Fellowship topic to other Fellows and UoM D4H staff.

As of March 2019, a total of 31 Fellows from 12 countries have completed a CRVS Fellowship, all of whom worked on topics across a wide range of CRVS interventions (Additional file 1). The topic areas have included measuring the outcomes of interventions to improve registration data quality and completeness, assessing the quality of medical certification of COD, analysing routine VA data to strengthen the evidence base of causes of community deaths, and developing country-specific integration modules to link data generated from VA with the existing CRVS system. One Fellow from the Ministry of Health in Peru demonstrated national improvements in the quality of COD data from the introduction of an online death certification system and training of doctors in standard death certification practices, identifying specific areas where further improvement in training could occur [22]. Another Fellow from the Cabinet Division in Bangladesh found an increased registration completeness in pilot areas due to a new death notification system, with complementary qualitative research identifying various successes and challenges associated with the pilot that have informed recommendations for the roll-out of the new death notification system [23].

Upon returning to their country, Fellows are expected to implement and transfer the knowledge and skills they learned into their daily work, and to become CRVS ‘champions’ to promote the benefits of CRVS systems as a primary source of evidence of population health. The Fellowship supervisor monitors the Fellow’s progress and provides technical advice to ensure the appropriate application of skills into CRVS activities. A notable outcome of the CRVS Fellowship Program has been the reported improvement in professional status, including respect and recognition, and confidence of Fellows once they return home – a significant benefit of being able to devote time to working on a specific topic away from their routine daily work and engagement with other Fellows and D4H specialists. Further, there are 21 documents publicly available about the fellowships of 31 Fellows, including 1 academic paper (with another 4 in development), 10 reports and 11 profiles of Fellows’ experiences [24].

Feedback from the fellows on the programme has been overwhelmingly positive, with comments such as:

“ *… This Fellowship changed me. It was an incredible experience. I have learned so much in these two months.*”

“*I had a great chance to study about qualitative methods through this Fellowship, and it allowed me to contribute towards filling a technical gap in our country. I was able to apply my new knowledge and skills related to qualitative interview methods as part of the mid-term evaluation of verbal autopsy implementation in* [country].”

“*The Data for Health Initiative has been very useful in opening our eyes to rework the way we’re doing things in* [country] *and I’m excited to go back to work and directly apply some of the knowledge I’ve gained during my time in the fellowship …* ”

“ *… this fellowship will not only benefit me as an individual but the entire country at large for improved CRVS implementation …* ”

## Conclusion

The Knowledge Gateway developed by the University of Melbourne as part of the D4H Initiative is an easily accessible, electronic repository of CRVS knowledge, experiences and challenges that will be a lasting resource for CRVS efforts in the future as the global movement gains momentum and more countries and development partners understand its importance. The complementary strategy to develop focused, comprehensive CRVS skills via in-country or regional training as well as the CRVS Fellowship Program provide the expertise and leadership in countries to effectively capitalise on these resources and contribute to the sustainability of country investment in CRVS. The suite of 10 CRVS courses included in the repository of learning materials can be applied in a wide range of countries to expand the skills and knowledge base required for monitoring and evaluation of CRVS scale-up in the future. Building capacity of local staff will help to ensure that they will build the acquired knowledge into their daily work, resulting in broader improvements in the quality of data generated by their CRVS systems. Having access to reliable and timely CRVS data is crucial for policymakers looking to develop relevant and responsive health policies and programmes, and building the confidence of the CRVS workforce will help countries continue to improve their CRVS systems long into the future.

## Supplementary information

**Additional file 1.** CRVS Fellows, Data for Health Initiative.

## Data Availability

Not applicable.

## Footnotes

1 Between March 2015 and March 2019, these countries were Bangladesh, Brazil, China (Shanghai), Colombia, Ecuador, Ghana, India (Mumbai), Malawi, Morocco, Myanmar, Papua New Guinea, Peru, Philippines, Rwanda, Solomon Islands, Sri Lanka, Tanzania and Zambia.

2 ANACONDA (Analysis of Causes of National Death for Action) is an electronic tool that assesses the accuracy and completeness of mortality and cause of death data by checking for potential errors and inconsistencies. ANACONDA is based on the guidelines for assessing the quality of mortality statistics that was developed at the Health Information Systems Knowledge Hub.
