# Supplementary material for: Addressing critical knowledge and capacity gaps to sustain CRVS system development
Source: BMC Med. 2020 Mar 9;18:46. doi: 10.1186/s12916-020-01523-y (PMC7061462; doi:10.1186/s12916-020-01523-y)
Supplement: Supplementary file 1 — Additional file 1. CRVS Fellows, Data for Health Initiative. [file 12916_2020_1523_MOESM1_ESM.docx]

**Additional File 1.** CRVS Fellows, Data for Health Initiative

| **Country** | **Organisation** | **Topic** | **Link to publication (if applicable)** |
| --- | --- | --- | --- |
| Bangladesh | Health | Quality of medical certification in four pilot hospitals: findings from the introduction of the international form of medical certificate of cause of death (COD) in Bangladesh | <https://crvsgateway.info/file/9786/2699> |
| Bangladesh | Health | Verbal autopsy in routine mortality surveillance in Bangladesh: CODs and lessons learned in conducting verbal autopsy (VA) interviews | <https://crvsgateway.info/file/10191/3060> |
| Bangladesh | Cabinet Division | Evaluation of the ‘Kaliganj Model’ for proactive birth and death notification and registration | <https://crvsgateway.info/file/10790/3207> |
| Brazil | Health | ANACONDA: Portuguese translation and improving data visualisations | <https://crvsgateway.info/file/9779/2667> |
| Brazil | Health | Investigation of garbage codes to improve COD statistics in Brazil | <https://crvsgateway.info/file/9783/1855> |
| Brazil | Statistics & Health (joint Fellowship) | Estimating the completeness of death reporting in Brazil using record linkage | <https://crvsgateway.info/file/9782/2631> |
| China, Shanghai | Health | Evaluation of the performance of the Shanghai Civil Registration and Vital Statistics (CRVS) system, 2002–2016 | <https://crvsgateway.info/file/9776/1525> |
| China, Shanghai | Health | Analysis of medical records review and VA data in Shanghai | <https://crvsgateway.info/file/10384/3133> |
| China, Shanghai | Health | SmartVA and ANACONDA improvement in China’s CRVS system | – |
| China, Shanghai | Health | Analysis of the quality of COD data in Shanghai | – |
| Ecuador | Statistics | Estimating the completeness of birth and death registration in Ecuador | <https://crvsgateway.info/file/9781/2484> |
| Ghana | Health | VA scale-up in Ghana | <https://crvsgateway.info/file/9787/2860> |
| Ghana | Health | Development of a VA management dashboard in Ghana | <https://crvsgateway.info/file/9789/1829> |
| Ghana | Births and Deaths Registry | e-Notification and registration of deaths in Ghana | – |
| Myanmar | Statistics | Experiences of midwives and public health supervisors (PHS-II) in the VA intervention in Myanmar | <https://crvsgateway.info/file/9780/2232> |
| Myanmar | Statistics | Analysis of the completeness of death registration and summary mortality statistics by province | – |
| Myanmar | Health | Analysis of CODs in Myanmar using VA | – |
| Papua New Guinea | Health | Quality assessment and analysis of Medical Certificates of COD from Papua New Guinea hospitals | <https://crvsgateway.info/file/9785/2376> |
| Peru | Health | Improving death certification in Peru: assessing the impact of two death certification interventions to improve COD data | <https://crvsgateway.info/file/9775/2435> |
| Peru | Statistics | Estimating coverage and omission of the register of births and deaths at the national and subnational level in Peru | – |
| Philippines | Health | Assessing the outcome of a physician training intervention on medical certification for COD in the Philippines | – |
| Philippines | Statistics | Assessing the quality and completeness of birth and death registration, and quality of COD data in the Philippines using ANACONDA | <https://crvsgateway.info/file/9777/2483> |
| Rwanda | Statistics | Monitoring notification and registration of vital events and system integration processes | <https://crvsgateway.info/file/9784/1977> |
| Rwanda | Health | Review and documentation of experiences in introducing routine VA as part of CRVS strengthening in Rwanda | <https://crvsgateway.info/file/10218/3059> |
| Rwanda | Statistics | Analysis of medical certification of COD and VA data | <https://crvsgateway.info/file/9788/2374> |
| Sri Lanka | Health | Developing a SmartVA DHIS2 integration platform that would streamline all manual CRVS data flow processes into a single automated process | <https://crvsgateway.info/file/9778/2805> (profile)  <https://crvsgateway.info/file/10032/3010> (report) |
| Sri Lanka | Health | Development and implementation of an e-learning module on medical certification of COD for doctors | – |
| Tanzania | Births and Deaths Registry | Measuring the performance of decentralization of birth registration to local government | – |
